# Novel rare genetic variants of familial and sporadic pulmonary atresia identified by whole-exome sequencing

**DOI:** 10.1515/biol-2022-0593

**Published:** 2023-05-19

**Authors:** Junyue Xing, Hongdan Wang, Yuanyuan Xie, Taibing Fan, Cunying Cui, Yanan Li, Shuai Wang, Weiyue Gu, Chengzeng Wang, Hao Tang, Lin Liu

**Affiliations:** Henan Key Laboratory of Chronic Disease Management, Central China Fuwai Hospital of Zhengzhou University, Fuwai Central China Cardiovascular Hospital & Central China Branch of National Center for Cardiovascular Diseases, Zhengzhou, Henan, 451464, China; National Health Commission Key Laboratory of Cardiovascular Regenerative Medicine, Heart Center of Henan Provincial People’s Hospital, Central China Fuwai Hospital of Zhengzhou University, Fuwai Central China Cardiovascular Hospital & Central China Branch of National Center for Cardiovascular Diseases, Zhengzhou, Henan, 451464, China; Medical Genetics Institute of Henan Province, Henan Provincial People’s Hospital, Zhengzhou University People’s Hospital, Zhengzhou 450003, China; National Health Commission Key Laboratory of Birth Defects Prevention, Henan Key Laboratory of Population Defects Prevention, Zhengzhou 450002, China; Department of Children’s Heart Center, Henan Provincial People’s Hospital, Department of Children’s Heart Center of Central China Fuwai Hospital, Henan Key Medical Laboratory of Tertiary Prevention and Treatment for Congenital Heart Disease, Central China Fuwai Hospital of Zhengzhou University, Zhengzhou, Henan, 451464, China; Department of Ultrasound, Fuwai Central China Cardiovascular Hospital, Central China Fuwai Hospital of Zhengzhou University, Zhengzhou, 451464, China; Department of Translational Medicine Center, Chigene (Beijing) Translational Medical Research Center Co., Beijing, 100176, China; Department of Ultrasound, The First Affiliated Hospital of Zhengzhou University, Zhengzhou, 450052, Henan, China; Department of Pediatrics, The Seventh Medical Center of Chinese PLA General Hospital, Beijing, 100700, China

**Keywords:** pulmonary atresia, whole-exome sequencing, rare variants, genetics

## Abstract

Pulmonary atresia (PA) is a severe cyanotic congenital heart disease. Although some genetic mutations have been described to be associated with PA, the knowledge of pathogenesis is insufficient. The aim of this research was to use whole-exome sequencing (WES) to determine novel rare genetic variants in PA patients. We performed WES in 33 patients (27 patient–parent trios and 6 single probands) and 300 healthy control individuals. By applying an enhanced analytical framework to incorporate de novo and case–control rare variation, we identified 176 risk genes (100 de novo variants and 87 rare variants). Protein‒protein interaction (PPI) analysis and Genotype-Tissue Expression analysis revealed that 35 putative candidate genes had PPIs with known PA genes with high expression in the human heart. Expression quantitative trait loci analysis revealed that 27 genes that were identified as novel PA genes that could be affected by the surrounding single nucleotide polymorphism were screened. Furthermore, we screened rare damaging variants with a threshold of minor allele frequency at 0.5% in the ExAC_EAS and GnomAD_exome_EAS databases, and the deleteriousness was predicted by bioinformatics tools. For the first time, 18 rare variants in 11 new candidate genes have been identified that may play a role in the pathogenesis of PA. Our research provides new insights into the pathogenesis of PA and helps to identify the critical genes for PA.

## 1 Introduction

Pulmonary atresia (PA) is a severe cyanotic congenital heart disease that accounts for 1.3–3.4% of congenital heart malformations [1,2]. Nonetheless, the incidence of PA in different populations varies [3]. The main feature of PA is the lack of connection between the ventricular artery and the pulmonary artery. Without surgical intervention, the early mortality rate is as high as 80%, and very few children can live to adulthood [4]. According to whether there is a ventricular septal defect, PA is divided into two types: PA with ventricular septal defect (PA-VSD) and PA with the intact ventricular septum (PA-IVS) [5,6,7]. The occurrence times of the two types of PA are distinct. Compared with PA-VSD, the damage from PA-IVS occurs in later pregnancy. PA-VSD damage occurs before the complete formation of the ventricular septum, while PA-IVS damage occurs after the completion of the ventricular septum formation [8]. PA infants usually have heart failure and cyanosis in the clinic. During the foetal period, prenatal ultrasonography has a high detection rate of PA-VSD, which is beneficial for the choice of surgical method. However, not all potential children can be detected by ultrasonography, especially those with main pulmonary collateral arteries [9]. At present, the main treatment for PA is still surgical treatment [10]. Although surgical techniques have been improved, the prognosis of PA is still poor [4].

Abnormal cardiac embryonic development is the main cause of congenital heart disease, so genetic research on congenital heart disease has always concentrated on the process of cardiac development. PA is a type of conic arterial trunk malformation, and its mechanism may be related to developmental abnormalities of the conic arterial trunk. Although the 22q11 microdeletion was found in approximately 8–17% of patients with conic arterial malformation associated with other systemic abnormalities, it rarely occurred in patients with isolated conic arterial malformation [11,12]. Through animal models, it was found that *Nkx2.5*, *Isl1*, *Foxh1*, *Foxc1*, *Fgf8*, *Tbx1*, and other genes played a role in the development, proliferation, and migration of cardiac neural crest cells and secondary heart field cells [13,14,15,16,17]. If these genes are deleted or mutated, conic arterial trunk malformation might occur [18]. The removal of the second heart area in HH14 chicken embryos led to PA, and the removal of the second heart area in HH18 chicken embryos led to coronary artery abnormalities [19]. Blocking the FGF signalling pathway in HH14 chicken embryos can also lead to PA. Nonetheless, the aforementioned studies are rarely verified in human heart development. Genetic evaluation of PA-IVS cases in identical twins demonstrated that *WFDC8* and *WFDC9* had a 55 kb deletion, but this was not found to be associated with genetic diseases, and the clinical significance was unclear [20]. Some PA candidate genes have been screened by whole-exome sequencing (WES). Fifty-six rare variations of seven new candidate genes (*DNAH10*, *DST*, *FAT1*, *HMCN1*, *HNRNPC*, *TEP1*, and *TYK2*) were screened in PA/VSD (*n* = 60) and PA/IVS (*n* = 20), and it was found that candidate genes of PA/VSD and PA/IVS were different. Three new rare copy number variations (CNVs), 16p11.2 del (*PPP4C*), 5q35.3 del (*FLT4*), and 5p13.1 del (*RICTOR*), were found in PA-VSD [21]. However, the function of these candidate genes still needs to be validated by further research [22].

The aforementioned PA studies suggest that many PA-related genes have not been found. With the development of next-generation sequencing technology, WES is increasingly being used in disease research, and it has gradually become a common screening method for molecular genetics research of diseases. In this study, to screen new PA pathogenic genes, WES was performed on peripheral blood samples of both 27 familial cases and 6 sporadic cases of PA. Bioinformatics screening was performed to determine the candidate genes to lay the foundation for exploring the molecular genetic pathogenesis of PA.

## 2 Materials and methods

### 2.1 Study population

Our cohort included 33 unrelated PA cases (27 familial and 6 sporadic cases) and 300 healthy control individuals (healthy children’s parents from the Chigene Chinese Gene-Phenotype Database) diagnosed by echocardiography, cardiac catheterization, or surgery. Enhanced association analysis was conducted by using two approaches: family-based de novo variant analysis and nonfamily rare variant association analysis (RVAS). The TADA-de novo model of TADA software was used to analyse the pathogenicity of de novo variants, and the significant top 100 genes are listed. EPACTS software was used for RVAS analysis of 33 cases and control samples from the Chigene Database. skat, b.callapse, b.madsen, and b.wcnt are gene-wise or group-wise tests of epacts software for gene-based effect analysis.

### 2.2 Sample collection

For the DNA extraction and whole-exome library construction, umbilical cord blood or foetal tissue genomic DNA was extracted using the Blood Genome Column Medium Extraction Kit (Kangweishiji, China) according to the manufacturer’s instructions. The extracted DNA samples were subjected to quality control using a Qubit 2.0 fluorimeter and electrophoresis with a 0.8% agarose gel for further protocols. Protein-coding exome enrichment was performed using xGenExome Research Panel v1.0 (IDT, Iowa, USA), which consists of 429,826 individually synthesized and quality-controlled probes. This targets a 39 Mb protein-coding region (19,396 genes) of the human genome and covers 51 Mb of end-to-end tiled probe space.


**Informed consent:** Informed consent has been obtained from all individuals included in this study.
**Ethical approval:** The research related to human use has been complied with all the relevant national regulations, institutional policies, and in accordance with the tenets of the Helsinki Declaration, and has been approved by the Ethics Committee of The First Affiliated Hospital of Zhengzhou University (2020-KY-142).

### 2.3 Sequencing

High-throughput sequencing was performed by an Illumina NovaSeq 6000 series sequencer (PE150), and not less than 99% of the target sequences were sequenced. The sequencing process was performed by Chigene.

### 2.4 Bioinformatics analysis

(1) For quality control, raw data were processed by fastp for adapter removal and low-quality read filtering.
(2) For SNV and indel screening, paired-end reads were aligned to the Ensemble GRCh37/hg19 reference genome. The results were sorted with SAMtools contrast, and the repeat sequence was marked with Picard. According to the analysis process of GATK, the mutation sites were identified to obtain the single nucleotide polymorphism (SNP) and the insertion or deletion (Indel) information of small fragments in each sample. All variation loci were compared with 1000Genome, ExAC, GnomAD, and other databases, and low-frequency variants and de novo variants were screened out.
(3) For CNV screening, adaptor sequences and reads of low quality were removed. Reads were mapped to reference genome hg19 using the BWA software. CNVs of 100 KB and greater in length were detected using Chigene independently developed software packages for CNV detection. Intervals of CNV were detected and screened according to public CNV databases. The CNV databases Decipher, ClinVar, OMIM, DGV, and ClinGen were used as references to annotate the pathogenic classification of each screened CNV.
(4) For variant annotation and pathogenicity prediction, the online system independently developed by Chigene was used to annotate database-based minor allele frequencies (MAFs) and ACMG practice guideline-based pathogenicity of every yielded gene variant, and the system also provided serial software packages for conservative analysis and protein product structure prediction. The databases for MAF annotation, including the dbSNP, ExAC_EAS, GnomAD_exome_EAS, Sift, Polypen2_hdiv, and MutationTaster software packages, were used to predict protein product structure variations. As a prioritized pathogenicity annotation to ACMG guidelines, the OMIM, HGMD, and ClinVar databases were used to confirm the pathogenicity of every variant. Since clinical sample data were used in this study, there was a hospital enrichment effect leading to selection bias in the results. In general, the size of the hospital enrichment effect is approximately 5–10 times. In this study, we selected a fivefold enrichment threshold for screening. The calculation method of the hospital enrichment effect divides the observed prevalence rate by the estimated prevalence rate ([case_sample_number/33]/[control_sample_number/300] > 5). To predict functional changes in variants at splicing sites, the MaxEntScan, dbscSNV, and GTAG software packages were used instead of the product structure prediction software. The protein sequences were downloaded from the UniProt website. The amino acid sequences of humans were compared with those of other species by MEGA 7 sequence alignment software. The change value of free energy (ΔΔG) was quantitatively calculated by I-Mutant to predict the effect of amino acid mutation on protein stability online. I-Mutant is free software for analysing the stability of the protein. ΔΔG is an energy prediction parameter that is used for quantitative calculation of the protein stability free energy difference caused by a single amino acid mutation. This reflects the effect of thermal denaturation of amino acid mutations on protein stability. The effect of a single mutation on the overall stability of a protein can be analysed based on the PDB ID or sequence primary structure information. If the protein tends to be stable due to mutation, then ΔΔG > 0; otherwise, the protein stability decreases after the mutation. The SWISS-MODEL online website was used to predict the protein structures of the wild type and mutant type.

### 2.5 Protein‒protein interaction (PPI) analysis

PPI data were extracted from the STRING database. The statistically significant candidate genes from de novo and RVAS and the known PA genes in the HPO (Human Phenotype Ontology) database and Phenolyzer database were combined with the STRING service platform, and the species was limited to *Homo sapiens* to construct the protein interaction network. The key PPI network was obtained with a confidence score ≥0.4 as the screening condition.

### 2.6 Genotype-tissue expression (GTEx)–expression quantitative trait loci (eQTL) analysis

Using the open-source tissue expression data provided by GTEx v8, the differential expression of candidate genes obtained by PPI analysis in related normal tissues (left ventricle and atrial appendage) was analysed, and genes with high expression in the heart were selected. eQTL analysis was executed by FastQTL software to correlate genotype and gene expression. Genes whose expression levels can be affected by surrounding SNPs were screened.

## 3 Results

### 3.1 Screening process of candidate genes

**Figure 1:**
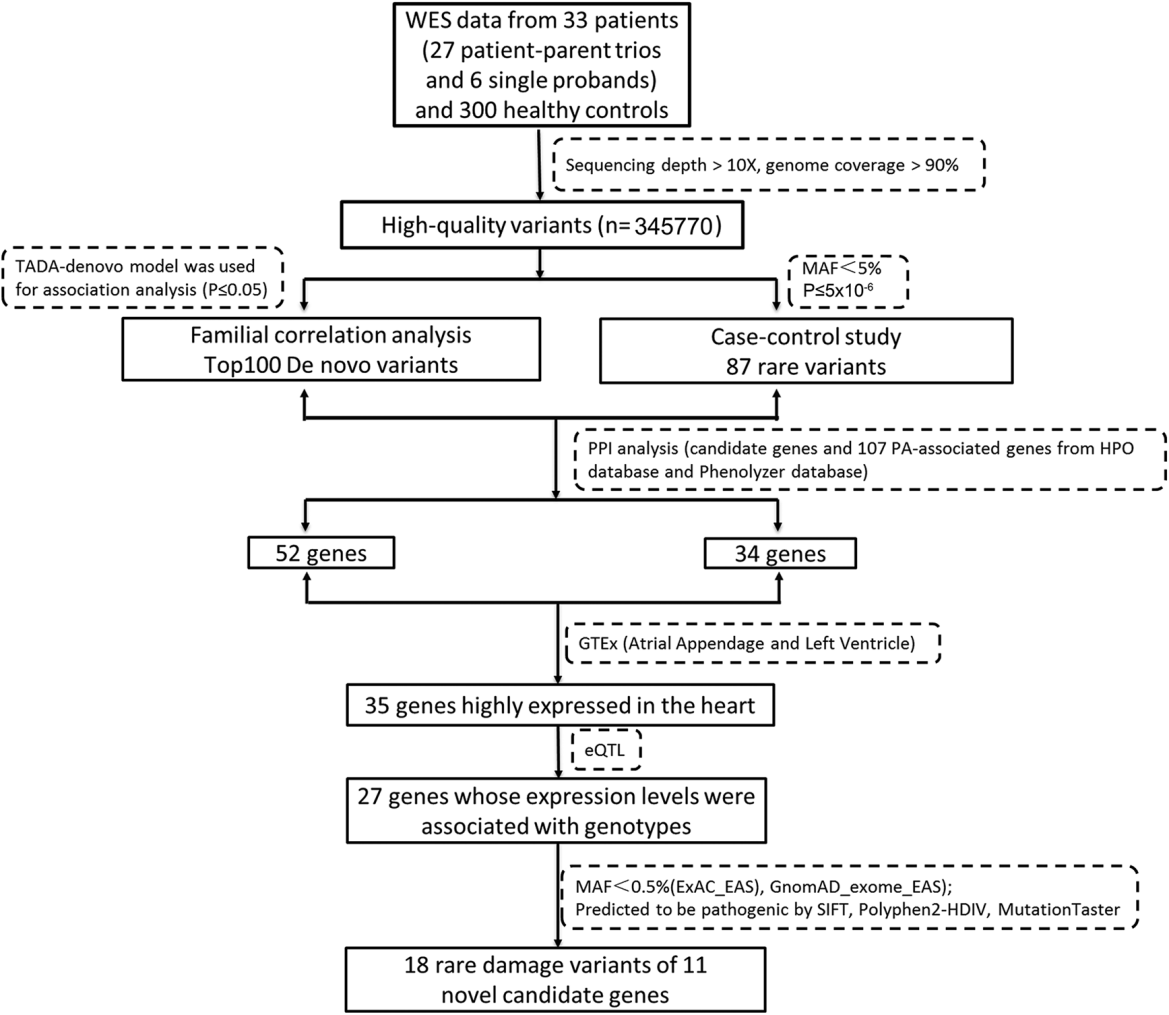
The workflow chart of the study. The workflow shows the analytical strategies for the different steps taken in WES analysis. After mutation invocation and annotation, candidate genes were collected by network analysis and gene expression analysis.

Pedigree-based de novo analysis and case‒control-based RVAS research strategies were adopted to evaluate the novel genetic risk genes of PA patients (Figure 1). As shown in Figure 1, through the analysis of WES data from 33 PA cases and 300 healthy control individuals, 345,770 highly reliable variations were successfully obtained. The TADA-De novo model was used for association analysis, and the top 100 significant genes were listed according to *P* ＜ 0.05 and at least one de novo missense variation of each gene (Table S1). Four models were used in RVAS research: b.collapse.epacts, b.madsen.epacts, b.wcnt.epacts, and skat.epacts. Eighty-seven rare variations of genes were screened out using MAF < 5% and *P* ≤ 5 × 10^−6^ by combining the genes analysed by four models (Table S2). Gene interaction network and expression analysis of GTEx indicated that 35 candidate genes highly expressed in the human heart had PPIs with known PA genes. There were 27 genes that could be affected by SNPs in the eQTL analysis. According to the ACMG standard guidelines, 18 rare pathogenic variations in 11 genes were screened out in the ExAC_EAS and GnomAD_exome_EAS databases with MAF < 0.5%.

### 3.2 Overall comparison of variants between the case and control samples

**Figure 2:**
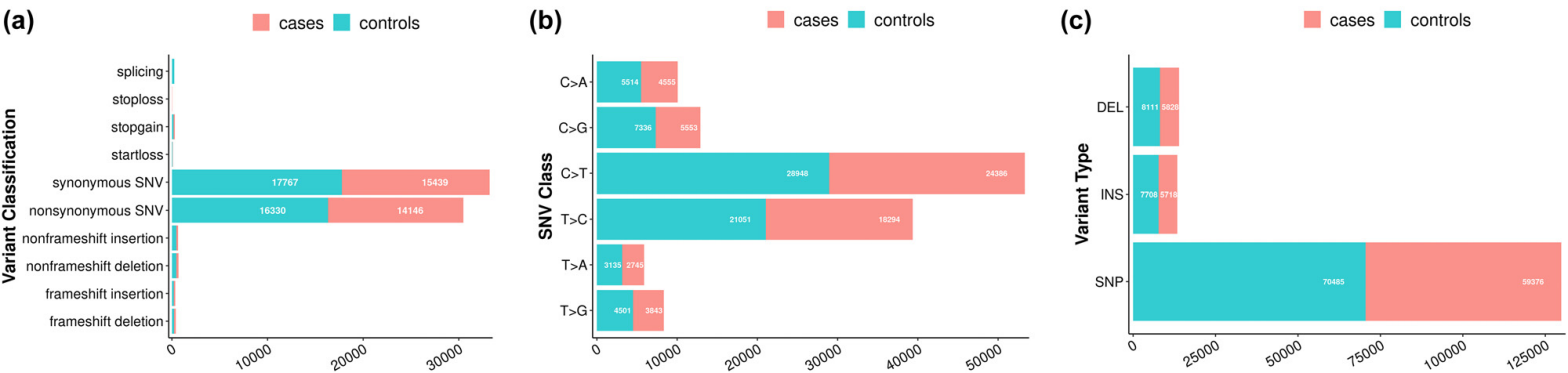
Comparisons of rare damaging variants between the case and control groups. The number of variants in each variant classification, SNV class, and variant type is presented in (a–c), respectively.

The variation classification, variation type, and variation number in the SNV class were statistically analysed (Figure 2). In the 59,376 nucleotide sites of the case samples, 42,680 transitional and 16,696 transversional sites with 5,828 indels were found, and the ratio between transition and transversion was 2.56. In the 70,485 nucleotide sites of the control samples, 49,999 transitional and 20,486 transversional sites with 8,111 indels were found, and the ratio between transition and transversion was 2.44. The ratio of transversional sites to transitional sites is greater than 2, indicating that base substitution is not affected by the saturation effect.

### 3.3 Gene networks

**Figure 3:**
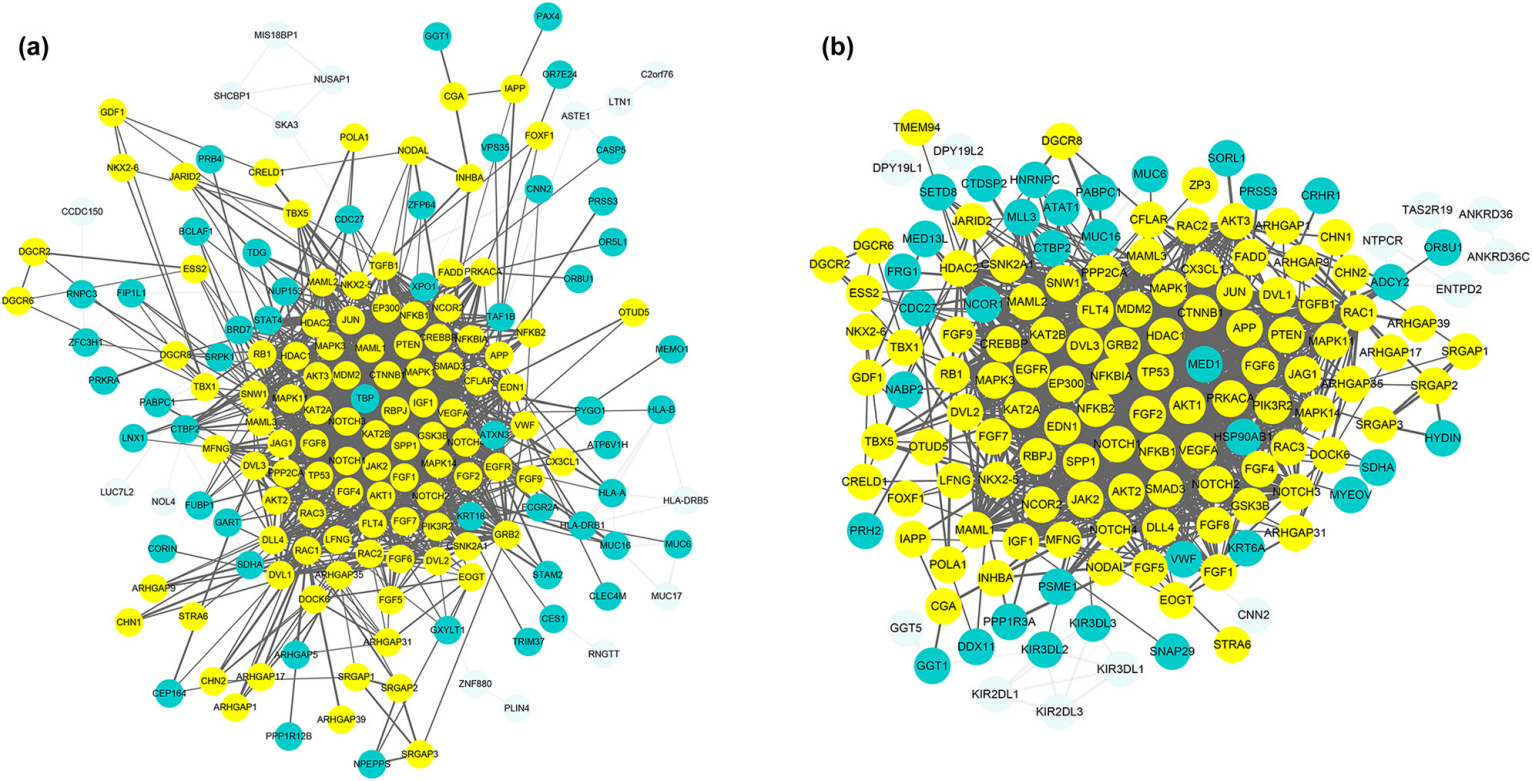
PPI network between known PA-associated genes (yellow nodes) and del candidate genes (blue nodes). Network interaction of proteins (confidence score ≥0.4) was performed by using Cytoscape, bioinformatics software with the STRING database. Fifty-two de novo variants (a) and 34 rare variants (b) interacting with known PA genes were screened out.

To determine the potential contribution of these pedigree-based and case–control-based candidate genes to PA, 107 known PA-related genes reported in the HPO and Phenolyzer databases were analysed (Table S3). The STRING tool was employed to obtain PPI relationships for the known PA genes. Using a confidence score of ≥0.4, the network of PPI relationships exhibited 78 key genes, including 52 de novo variants (Figure 3a) and 34 rare variants (Figure 3b) (Table S4). Except for the VWF gene, the other screened genes were identified as novel PA genes.

### 3.4 Correlation analysis between variants and expression levels

GTEx is a database with tissue-specific gene expression and regulation. Samples from 54 different parts of the human body were simultaneously subjected to transcriptome sequencing and genotyping analysis [23]. The process by which DNA mutations affect mRNA expression is called eQTL analysis. The expression levels of all samples were analysed by the GTEx database, and 35 highly expressed candidate genes in the heart (atrial appendage and left ventricle) were screened (Table S5, Figure S1). The eQTL analysis was performed by FastQTL software to correlate genotype with gene expression. Twenty-seven genes that were identified as novel PA genes that could be affected by the surrounding SNP were screened (Table S5).

### 3.5 Hospital enrichment effect filtration and pathogenicity analysis of rare variants

Rare variants were screened in the ExAC and gnomAD databases of the East Asian population with a threshold of MAF <0.5%. We selected fivefold for filtering hospital enrichment effect filtration. The SIFT, Polyphen2, and MutationTaster databases were used to analyse the harmfulness of rare mutations. Finally, we screened 18 harmful rare variants in 11 genes (Table S6). These mutation sites have not been reported in the literature and are not included in the dbSNP database.

### 3.6 Protein structure prediction analysis

Most mutations have ΔΔG < 0, which are unstable mutations. Mutant proteins with ΔΔG < 1 showed significant instability. The ΔΔG of the BCLAF1 p.R271S, PABPC1 p.L562S, and CTBP2 p.G117S gene mutation sites were all less than 1, indicating that the protein stability was significantly decreased (Table S7). The amino acid mutations of succinate dehydrogenase-flavin (SDHA), BCLAF1, PABPC1, CTBP2, and CNN2 are located in highly conserved regions (Figure S2). Usually, mutations in highly conserved regions are pathogenic.

**Figure 4:**
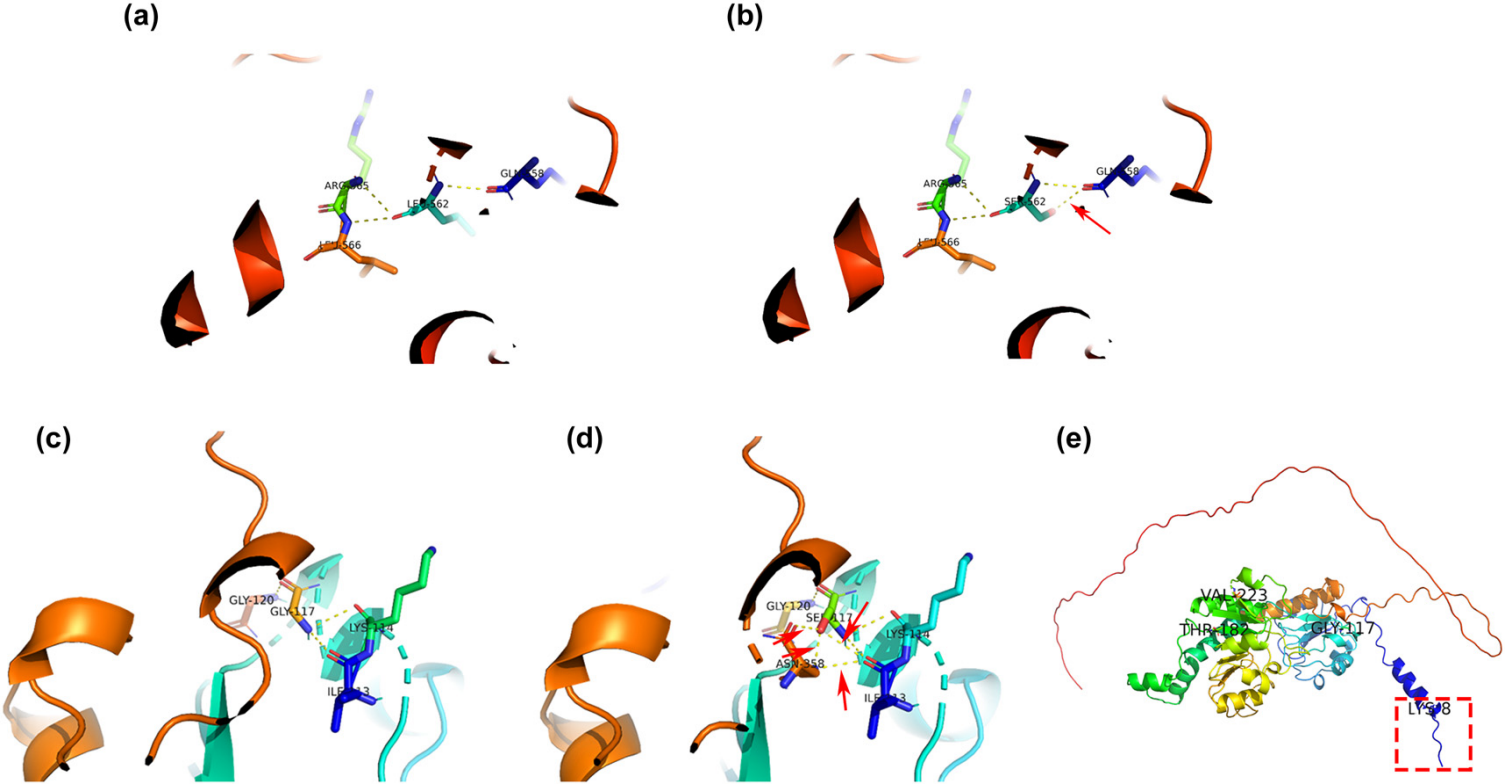
The structure prediction of the wild-type proteins and variants of PABPC1 and CTBP2. (a) Crystal structure of wild-type PABPC1. Crystal structure data were taken from RSCB PDB, I.D.: 1G9L. (b) Putative structure of the PABPC1 p.L562S mutant. A hydrogen bond was added between Ser562 and Gln558 (red arrow). (c) Crystal structure of wild-type CTBP2. Crystal structure data were taken from RSCB PDB, I.D.: 2OME. (d) Putative structure of the CTBP2 p.G117S mutant. Four hydrogen bonds have been added between Ser117, Ile113, Lys114, Gly120, and Asn358 (red arrows). (e) Putative structure of the CTBP2 p.K8X mutant.

The number of hydrogen bonds in PABPC1 and CTBP2 changed (Figure 4). PABPC1 p.L562S was found in three patients, and mutations of this site were not found in the healthy group. The amino acid changes from leucine to serine, and the mutant has an additional hydrogen bond. CTBP2 p.G117S was found in eight patients, and this site did not change in the healthy group. The mutant had an additional four hydrogen bonds. CTBP2 p. K8X stopped translation, resulting in a complete loss of the functional domain. This mutation was found in 17 patients, but not in the healthy control group. The physicochemical properties of side-chain radical of BCLAF1 p.R271S, BCLAF1 p.Q165K, PABPC1 p.L562S, CTBP2 p.T182M, CTBP2 p.G117S, and CNN2 p.R266Q changed.

## 4 Discussion

To explore the potential risk genes for PA, WES was performed for 33 patients and 300 healthy control individuals. For the first time, 18 rare damage variations in 11 new candidate genes were found to play a role in the pathogenesis of PA. This study expanded the range of PA pathogenic genes and provided new evidence for molecular genetics research on PA.

In mammalian embryonic development, the heart is the first organ to complete development. Abnormal embryonic development is the main cause of congenital heart disease. Studies have discovered that at the early stage of embryonic development, the heart needs to activate a large number of mRNAs for rapid division and differentiation. The expression of genes related to early embryonic development in mammals is primarily regulated at the post-transcriptional level [24]. The development of the heart valve starts with the endothelial-to-mesenchymal transition (EndoMT). First, some endothelial cells in the atrioventricular canal and outflow tract migrate to the cardiac glial area through the EndoMT to form mesenchymal cells. Subsequently, these mesenchymal cells highly proliferate and fuse to form a dense endocardial cushion with a cellular structure. Finally, the endocardial cushion is reshaped to produce an extracellular collagen matrix and form a mature valve [25,26,27]. Some transcription factors specifically highly expressed in the heart may be involved in the process of endothelial mesenchymal cell transformation, such as the *GATA* family (zinc finger protein transcription factor). *GATA4* and *GATA6* are highly expressed during endocardial cushion formation [28]. A mouse model was used to show that the expression of *GATA4* is necessary for the transformation of endothelial–mesenchymal cells in the atrioventricular endocardial cushion of mice [29]. *SDHA*, *BCLAF1*, *PABPC1*, *CTBP2,* and *CNN2* were found to be closely related to heart development.

PABPC1 p.L562 is located in the poly(A)-binding protein (PABP) functional domain. PABP is a class of highly conserved RNA-binding proteins that are ubiquitous in eukaryotes and can specifically recognize and bind to polyadenosine sequences. Eukaryotic proteins play an essential role in gene expression by binding to the 3′-poly(A) tail of mRNA. Although they lack catalytic activity, they can provide scaffolds in cells that bind to poly(A) tails and interact with other proteins to mediate transcription and translation and transport, and regulate mRNA degradation. PABPC1 is largely involved in a variety of post-transcriptional regulations to regulate gene expression, such as the regulation of mRNA translation initiation and microRNA-mediated expression. This post-transcriptional regulation of gene expression can regulate protein synthesis without changing transcriptional activity, allowing cells to respond quickly to external signals. Dynamic changes in poly(A) tail length can regulate the translation of specific mRNAs [30]. When the mRNA leaves the nucleus with a poly(A) tail of 250–300 A bases, the length gradually shortens and begins to extend when the mRNA is reactivated [31].

Increased expression of PABPC1 in cardiomyocytes can induce physiological cardiac hypertrophy [32]. In the hearts of adults and mice, when the length of the poly(A) tail was shortened, the expression level of PABPC1 decreased. When heart disease occurred, the poly(A) tail became longer and the expression level of PABPC1 increased. This indicates that PABPC1 regulates its own mRNA translation through the poly(A) tail [32]. The expression level of PABPC1 is low during early egg formation in *Xenopus laevis*. The expression level of PABPC1 increased rapidly after blastocyst transformation to early embryonic development, and PABPC1 was still found in the heart, brain, and other organs of adult *Xenopus laevis* [24]. Studies using model biological organisms, such as *Drosophila*, *Caenorhabditis elegans*, *Saccharomyces cerevisiae*, and *Xenopus laevis*, have identified that mutations of PABPC1 can lead to multiple organ malformations, including cardiac malformations [33]. Previous studies have demonstrated that *PABPC1* plays an important role in cardiac differentiation and development.

CTBP2 p.G117 is located in the d-isomer-specific 2-hydroxydehydrogenase catalytic domain (2-Hacid_dh) of the CTBP2 protein. The NAD binding domain is the largest component of the 2-hydroxydehydrogenase catalytic domain of the CTBP2 protein. CTBP2 p.K8X terminated the translation at the eighth amino acid, and the protein functional domain was completely lost. C-terminal binding protein (CTBP) was initially identified as a protein that interacts with C-terminal elements and is highly conserved in eukaryotes [34]. CTBP family proteins are mainly used as transcriptional corepressors. dCTBP (the drosophila homologue) was shown to function as a transcriptional corepressor. CTBP can also activate the transcription process [35,36]. A *CTBP2*-knockout mouse experiment showed that the expression of the Wnt3A target gene Brachyury in E10.5 embryos was significantly lower than that in E9.5 embryos. This indicates that *CTBP2* may be a Wnt-mediated transcriptional activator of Brachyury [37]. *CTBP2* plays a unique regulatory role in the development of mice. The lack of CTBP leads to the inhibition of the activity of a large number of transcription factors, which leads to defects in cardiac morphology in mice. Cardiac embryos show pericardial dilation, and the development of the heart tube is blocked [37].

The p.R271S and p.Q165K variants of BCLAF1 are located in the THRAP3-BCLAF1 functional domain. BCLAF1 (Bcl-2-related transcription factor) is a multifunctional protein with a DNA binding domain and RS functional domain rich in arginine-serine. *BCLAF1* is involved in the regulation of transcription and post-transcriptional processing. It can be used as a transcription factor and plays an important role in mRNA precursor splicing [38,39,40]. *BCLAF1* plays an important role in the growth and development of mice. *BCLAF1*-deficient mice did not show embryonic death [41] but exhibited arrested lung development, resulting in death shortly after birth [42]. BCLAF1 is a harmful molecule in cardiac I/R injury. Cardiomyocyte-specific overexpression of BCLAF1 aggravated cardiac I/R injury, while partial knockout of *BCLAF1* alleviated cardiac I/R injury. This is the first evidence of the underlying mechanism of *BCLAF1* in cardiac I/R injury and may indicate its role as a potential therapeutic target for ischaemic heart disease [43].

The p.V446A and p.A449V residues of SDHA are located in the FAD binding domain. Succinate dehydrogenase (SDH), also known as succinate ubiquinone oxidoreductase or mitochondrial complex II, is the only multisubunit enzyme that integrates into the inner membrane of mitochondria in the tricarboxylic acid (TCA) cycle. SDH plays an important role in TCA and the aerobic respiratory chain [44]. Mutations in SDH cause the disruption and overflow of electron transfer from mitochondrial complex II [45] and even reverse the transfer process of succinic acid to fumaric acid in TCA, which may be the basis of multisystem lesions [46]. It has been reported that when SDH mutations occur, the respiration ability of mitochondria completely disappears, and a large number of reactive oxygen species (ROS) are produced, leading to the death of cardiac myocytes [47]. SDHA is the only active group in the SDH structure and is the key factor in the TCA cycle and aerobic respiration chain. SDHB, SDHC, and SDHD are inactive groups that only play a role in maintaining structural stability [48]. As a key enzyme in mitochondria, SDHA is involved in energy metabolism. At normal oxygen levels, hypoxia-inducing factor (HIF-1α) is a major regulator of cell metabolism. The *SDHA* gene has a strong ability to balance selection recognition, and *SDHA* mutants regulate cellular oxygen balance by influencing it. *SDHA* gene mutations decrease SDH activity, resulting in succinic acid accumulation. HIF-1α hydroxylation is accompanied by the conversion of α-ketoglutaric acid to succinic acid. Additionally, the accumulation of succinic acid can inhibit the hydroxylation and degradation of HIF-1α, resulting in an increase in HIF-1α protein and its target gene expression. Increasing succinic acid in the mitochondria by succinic dehydrogenase drives more ROS production. The *SDHA* c.1351C > T mutation has been reported in cases of mitochondrial metabolic disorders accompanied by cardiac damage [49]. Cardiomyopathy associated with *SDHA* defects has been reported in neonates. Rare cases of autosomal recessive neonatal isolated dilated cardiomyopathy are associated with the SDHA G555E mutation [50].

There are four noncovalent forces (hydrogen bonds, hydrophobic forces, electrostatic forces, and van der Waals forces) and two covalent forces (sulphide bonds and metal ion binding forces) that maintain protein structure. The amino acid composition of a protein is related to its thermal stability. Hydrogen bonds are a strong force existing in the peptide chain and generally exist in proteins. Aliphatic amino acids (such as alanine, isoleucine, leucine, and valine) are mostly embedded in the protein, and the internal conformation stability of the protein is improved through hydrophobic interactions within the protein, thereby improving its temperature tolerance. Ionizable residues (such as arginine, aspartate, histidine, glutamic acid, and lysine) mainly maintain the stability of the external structure of the protein by forming a charge force, thereby promoting the overall stability of the protein. The interaction of aromatic amino acids (such as phenylalanine, tryptophan, and tyrosine) includes the interaction between aromatic groups and cation-π interactions, which plays an important role in stabilizing proteins.

The number of hydrogen bonds between PABPC1 and CTBP2 changed. PABPC1 p.L562 S changed from leucine (hydrophobic amino acid) to serine (polar noncharged amino acid), and one hydrogen bond was added. CTBP2 p.G117S added four hydrogen bonds. There were no hydrogen bond changes at other mutant sites, but the polarity of side chain groups at almost all mutant sites of BCLAF1, PABPC1, CTBP2, and CNN2 changed. Mutated at sites 446 and 449 of SDHA, alanine and valine residues were replaced with each other. The side chain of valine (isopropyl) is larger than that of alanine (methyl), and both are hydrophobic amino acids, which are generally distributed in protein molecules. Specifically, if the mutation is distributed in the active site, it can change the structure of the active site and may cause the loss of protein activity. The protein stabilities of BCLAF1, PABPC1, and CTBP2 were significantly decreased. It can be inferred that the functional domains of PABPC1 and CTBP2 play an important role in the occurrence of PA.

## 5 Conclusion

In this study, 18 novel mutations in 11 genes were discovered by WES and bioinformatics prediction analysis. These mutations have not been reported in the literature, and the dbSNP database is not included. Along with the structure of proteins, the relationship between the functional domains of new mutation sites and PA was analysed and discussed. The potential role of these novel mutation sites in the transcriptional regulation of cardiac embryonic development is worth exploring. This research study expanded the PA pathogenic gene spectrum and laid a foundation for the translation of basic medicine concepts for clinical genetic counselling, prenatal diagnosis, and gene therapy.

## Supplementary Material

Supplementary material
