# Supplementary material for: Novel rare genetic variants of familial and sporadic pulmonary atresia identified by whole-exome sequencing
Source: Open Life Sci. 2023 May 19;18(1):20220593. doi: 10.1515/biol-2022-0593 (PMC10199322; doi:10.1515/biol-2022-0593)
Supplement: Supplementary material [file biol-2022-0593-sm.pdf]

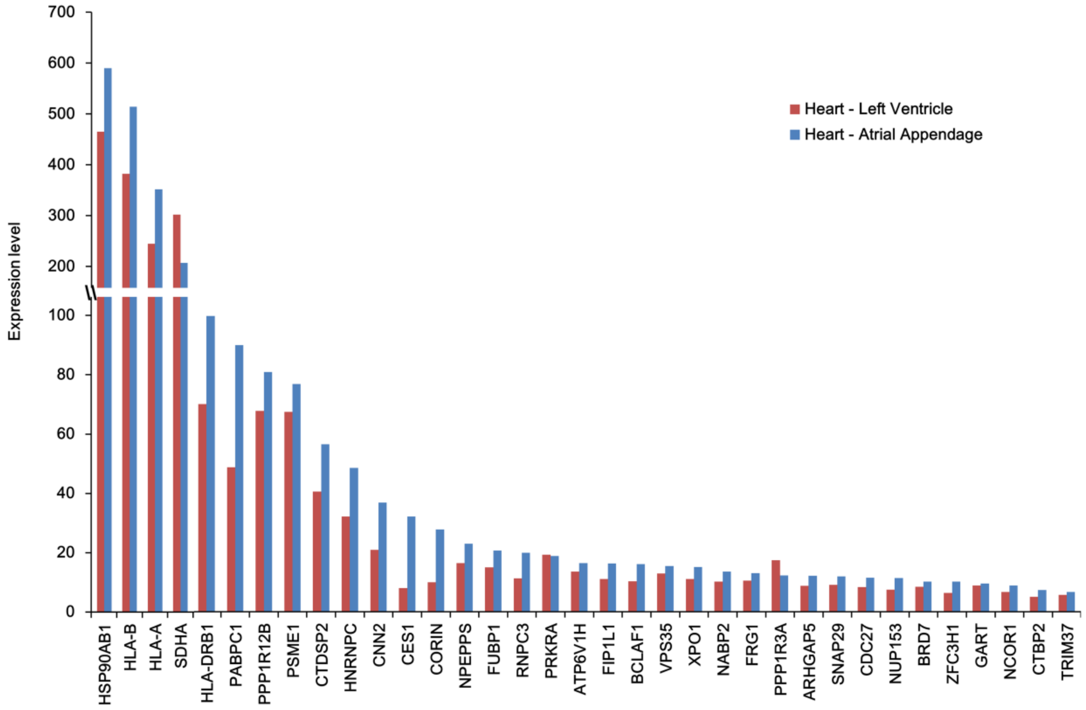

Supplementary Figure S1. Thirty-five candidate genes highly expressed in human heart. Transcriptomic Analyses for Identification and Prioritization of 35 candidate genes, highly expressed in left ventricle and atrial appendage. The mean expression levels of the differentially expressed lncRNAs for left ventricle and atrial appendage from the Genotype-Tissue Expression (GTEx) project.

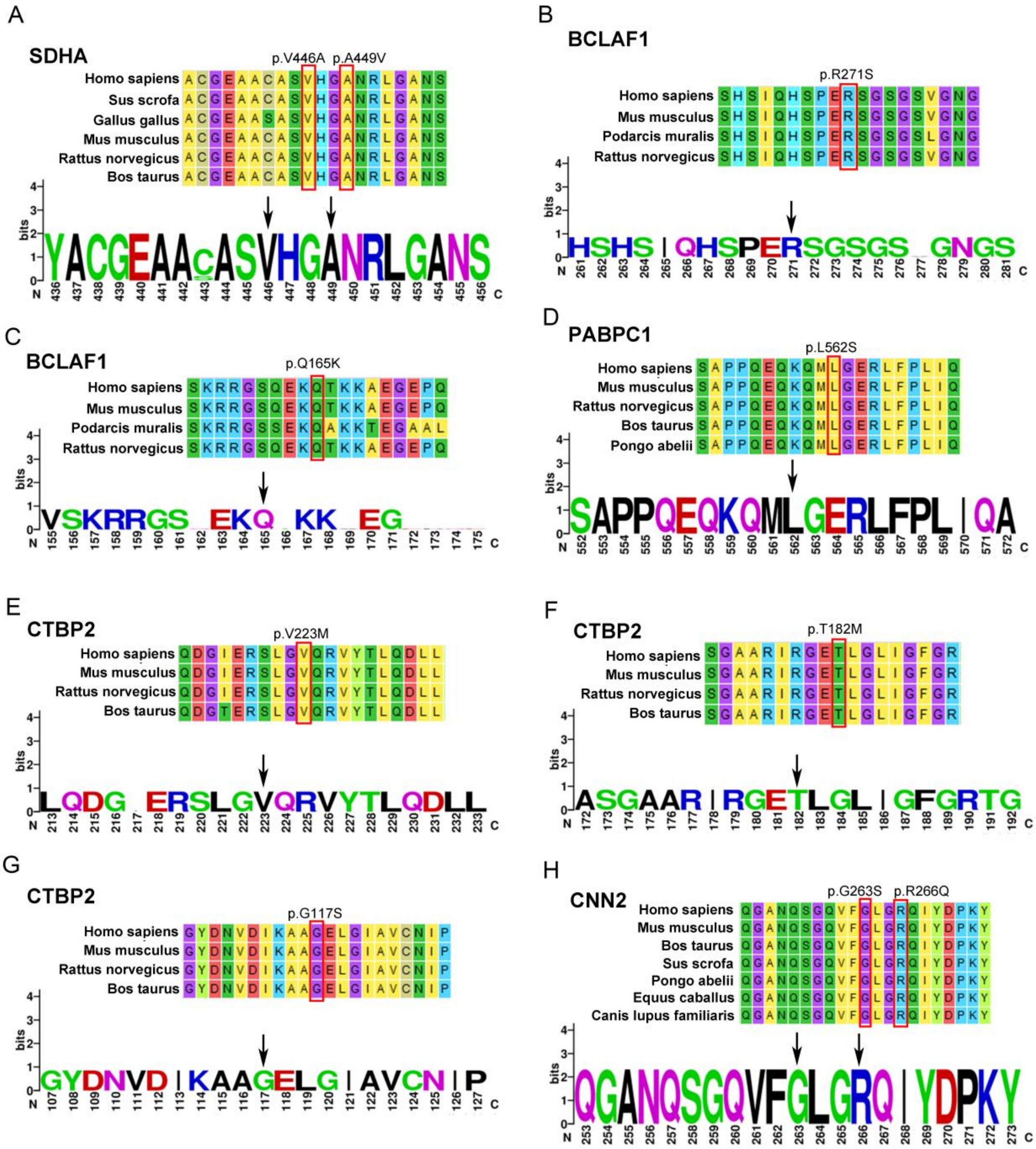

Supplementary Figure S2. Conservation of rare variants. High conservation of 10 rare variants across different species is shown according to the comparison of amino acid sequences.

Table S1
Gene list of de novo variants

de novo variants		
ANKMY2	MIS18BP1	TRIM77
AP3S1	MTCH2	TSHZ1
AQP7	MUC16	USP31
ARHGAP5	MUC17	VPS35
ASTE1	MUC6	WDSUB1
ATP6V1H	NOL4	XPO1
ATXN3	NPEPPS	ZFC3H1
BCLAF1	NUFIP1	ZFP64
BRD7	NUP153	ZNF280D
C15orf40	NUSAP1	ZNF880
C2orf76	OR5L1	
CACNA1B	OR7E24	
CASP5	OR8U1	
CCDC150	PABPC1	
CCDC30	PABPC3	
CDC27	PAX4	
CEP164	PCDHGA10	
CES1	PIBF1	
CHST15	PLIN4	
CLEC4M	PPP1R12B	
CNN2	PRAMEF2	
CORIN	PRB4	
CTBP2	PRKRA	
DNAJC11	PRSS3	
DZANK1	PYGO1	
FAM186A	RBMS1	
FCGR2A	RNF145	
FIP1L1	RNGTT	
FMNL2	RNPC3	
FUBP1	RP1L1	
GART	SDHA	
GBP3	SHCBP1	
GGT1	SIRPB1	
GXYLT1	SKA3	
HLA-A	SLC35F5	
HLA-B	SLC9B1	
HLA-DRB1	SRPK1	
HLA-DRB5	STAM2	
KRT18	STAT4	
KRTAP4-7	TAF1B	
LMBRD2	TAS2R30	
LNX1	TBP	
LTN1	TDG	
LUC7L2	TMBIM4	
MEMO1	TRIM37	

Table S2

Gene list of rare variants

b.collapse. epacts	b.madsen. epacts	b.wcnt. epacts	skat.epacts	**total rare variants**
AC008734.2	AC008734.2	AC008734.2	AC008734.2	AC008734.2
AGAP5	ACSM5	CCDC144NL	ACSM5	ACSM5
ANKRD18B	AGAP5	CTDSP2	ADCY2	ADCY2
ANKRD36C	ANKRD18B	FRG1	AGAP5	AGAP5
CBWD6	ANKRD36C	FRG1B	ANKRD18B	ANKRD18B
CCDC144NL	ATAT1	GGT1	ANKRD23	ANKRD23
CDC27	CBWD6	IGSF3	ANKRD36	ANKRD36
CNN2	CCDC124	KIR2DL1	ANKRD36C	ANKRD36C
CTBP2	CCDC144NL	MST1P9	ATAT1	ATAT1
CTDSP2	CDC27	MUC6	C6orf118	C6orf118
DHRS4	CNN2	NCOR1	CBWD6	CBWD6
DPY19L1	CRHR1	PCMTD1	CCDC124	CCDC124
FRG1	CTBP2	POTEC	CCDC144NL	CCDC144NL
GGT1	CTDSP2	SDHA	CDC27	CDC27
HYDIN	DHRS4	SETD8	CEP89	CEP89
IGSF3	DPY19L1	TEKT4	CNN2	CNN2
KIR2DL1	FRG1	TREML2	CRHR1	CRHR1
KRT6A	FRG1B	ZP3	CTBP2	CTBP2
KRTAP5-7	GGT1		CTDSP2	CTDSP2
MST1P9	HYDIN		DDX11	DDX11
NCOR1	IGSF3		DHRS4	DHRS4
PCMTD1	KIR2DL1		DPY19L1	DPY19L1
POTEC	KIR2DL3		DPY19L2	DPY19L2
PRSS3	KIR3DL1		ENTPD2	ENTPD2
SETD8	KIR3DL2		FRG1	FRG1
TAS2R31	KIR3DL3		FRG1B	FRG1B
TEKT4	KRT6A		GGT1	GGT1
TREML2	KRTAP5-7		GGT5	GGT5
ZP3	MST1P9		HNRNPC	HNRNPC
	NABP2		HSP90AB1	HSP90AB1
	NCOR1		HYDIN	HYDIN
	OR8U1		IGSF3	IGSF3
	PABPC1		KDM4E	KDM4E
	PCMTD1		KIAA1244	KIAA1244
	POMZP3		KIR2DL1	KIR2DL1
	POTEC		KIR2DL3	KIR2DL3
	PRSS3		KIR3DL1	KIR3DL1
	PVRIG		KIR3DL2	KIR3DL2
	RP11-14C22.5		KIR3DL3	KIR3DL3
	RP11-82O18.1		KRT6A	KRT6A
	SETD8		KRTAP5-7	KRTAP5-7
	SUSD5		KRTAP9-2	KRTAP9-2
	TAS2R19		LILRA6	LILRA6
	TAS2R31		MED1	MED1
	TEKT4		MED13L	MED13L
	TREML2		MLL3	MLL3
	ZNF492		MST1P9	MST1P9
	ZP3		MUC16	MUC16
			MUC6	MUC6
			MYEOV	MYEOV
			NABP2	NABP2
			NCOR1	NCOR1
			NTPCR	NTPCR
			OR8U1	OR8U1
			PABPC1	PABPC1
			PABPC3	PABPC3
			PCMTD1	PCMTD1
			POMZP3	POMZP3
			POTEC	POTEC
			PPP1R3A	PPP1R3A
			PRAMEF11	PRAMEF11
			PRH2	PRH2
			PRSS3	PRSS3
			PSME1	PSME1
			PVRIG	PVRIG
			RHPN2	RHPN2
			RP11-14C22.5	RP11-14C22.5
			RP11-82O18.1	RP11-82O18.1
			SDHA	SDHA
			SETD8	SETD8
			SLC4A11	SLC4A11
			SNAP29	SNAP29
			SORL1	SORL1
			SULT1A2	SULT1A2
			SUSD5	SUSD5
			TAS2R19	TAS2R19
			TAS2R31	TAS2R31
			TAS2R46	TAS2R46
			TEKT4	TEKT4
			THAP2	THAP2
			TMEM128	TMEM128
			TREML2	TREML2
			VWF	VWF
			ZNF492	ZNF492
			ZNF644	ZNF644
			ZNF676	ZNF676
			ZP3	ZP3

Table S3

Known PA-related genes reported in the HPO and Phenolyzer databases

HPO database	Phenolyzer database			
ARHGAP31	AKT1	EOGT	MAML3	SNW1
CRELD1	AKT2	EP300	MAPK1	SPP1
DGCR2	AKT3	ESS2	MAPK11	SRGAP1
DGCR6	APP	FADD	MAPK14	SRGAP2
DGCR8	ARHGAP1	FGF1	MAPK3	SRGAP3
DLL4	ARHGAP17	FGF2	MDM2	STRA6
DOCK6	ARHGAP31	FGF4	MFNG	TBX1
DVL3	ARHGAP35	FGF5	MMP21	TBX5
EOGT	ARHGAP39	FGF6	NCOR2	TGFB1
ESS2	ARHGAP9	FGF7	NFKB1	TMEM94
FADD	CFLAR	FGF8	NFKB2	TP53
FLT4	CGA	FGF9	NFKBIA	VEGFA
FOXF1	CHN1	GDF1	NKX2-5	VWF
GDF1	CHN2	GRB2	NOTCH1	
MMP21	CREBBP	GSK3B	NOTCH2	
NKX2-5	CRELD1	HDAC1	NOTCH3	
NKX2-6	CSNK2A1	HDAC2	NOTCH4	
NODAL	CTNNB1	IAPP	PIK3R2	
NOTCH1	CX3CL1	IGF1	PKD1L1	
OTUD5	DGCR2	INHBA	PPP2CA	
PIGL	DGCR6	JAG1	PRKACA	
PKD1L1	DGCR8	JAK2	PSAP	
POLA1	DLL4	JARID2	PTEN	
RBPJ	DOCK6	JUN	RAC1	
STRA6	DVL1	KAT2A	RAC2	
TBX1	DVL2	KAT2B	RAC3	
TMEM94	DVL3	LFNG	RB1	
	EDN1	MAML1	RBPJ	
	EGFR	MAML2	SMAD3	

Table S4

Genes with strong interactions with known PA genes

PPI-de novo variants	PPI-rare variants
ARHGAP5	ADCY2
ATP6V1H	ATAT1
ATXN3	CDC27
BCLAF1	CRHR1
BRD7	CTBP2
CASP5	CTDSP2
CDC27	DDX11
CEP163	FRG1
CES1	HNRNPC
CGR2A	HSP90AB1
CLEC4M	HYDIN
CNN2	KIR3DL2
CORIN	KIR3DL3
CTBP2	KRT6A
FIP1L1	MED1
FUBP1	MED13L
GART	MLL3
GGT1	MUC16
GXYLT1	MUC6
HLA-A	MYEOV
HLA-B	NABP2
HLA-DRB1	NCOR1
KRT18	OR8U1
LNX1	PABPC1
MEMO1	PPP1R3A
MUC16	PRH2
MUC6	PRSS3
NPEPPS	PSME1
NUP153	SDHA
OR5L1	SETD8
OR7E24	SNAP29
OR8U1	SORL1
PABPC1	VWF
PAX4	ZP3
PPP1R12B	
PRB4	
PRKRA	
PRSS3	
PYGO1	
RNPC3	
SDHA	
SRPK1	
STAM2	
STAT4	
TAF1B	
TBP	
TDG	
TRIM37	
VPS35	
XPO1	
ZFC3H1	
ZFP64	

Table S5

Candidate genes highly expressed in heart (heart ear and left ventricle); association analysis of genotype and gene expression

Gene	Heart-Atrial Appendage (TPM>5)	Heart-Left Ventricle (TPM>5)	eQTL
ARHGAP5	12.1009	8.67963	Yes
ATP6V1H	16.334	13.5072	Yes
BCLAF1	15.9866	10.3036	Yes
BRD7	10.1912	8.41523	Yes
CDC27	11.451	8.30196	Yes
CES1	32.0601	7.9969	Yes
CNN2	36.8001	20.8163	Yes
CORIN	27.7011	9.95325	Yes
CTBP2	7.32224	5.00914	Yes
CTDSP2	56.4321	40.4946	No
FIP1L1	16.2783	11.0628	Yes
FRG1	12.9814	10.4433	No
FUBP1	20.6829	14.9288	Yes
GART	9.53911	8.82876	Yes
HLA-A	351.535	244.471	Yes
HLA-B	513.843	382.314	Yes
HLA-DRB1	99.6645	69.9643	Yes
HNRNPC	48.4694	32.1273	Yes
HSP90AB1	589.883	465.045	No
NABP2	13.4942	10.1425	No
NCOR1	8.77854	6.62791	No
NPEPPS	22.8902	16.3755	Yes
NUP153	11.3718	7.4259	Yes
PABPC1	89.9248	48.6769	Yes
PPP1R12B	80.833	67.6926	Yes
PPP1R3A	12.2099	17.303	No
PRKRA	18.7682	19.1562	Yes
PSME1	76.739	67.3931	No
RNPC3	19.8169	11.2005	Yes
SDHA	206.773	301.984	Yes
SNAP29	11.9032	9.08668	No
TRIM37	6.6252	5.71911	Yes
VPS35	15.426	12.9093	Yes
XPO1	15.0695	10.9639	Yes
ZFC3H1	10.0979	6.29571	Yes

Table S6

Mutation site information of PA-related candidate genes

Gene Sym	Chr	Ref Allele	Seq Allele	NA Change[a]	AA Change[b]	ExAC_EAS[c]	GnomAD_exome_EAS	SIFT	Polyphen2_HDIV	Mutation Taster	dbSNP[d]	number of samples in case	number of samples in control
PRKRA	2q31.2	TAAAGAAATGTGGTTCTCTGGAGAAATATTACTAAATTTGGCAAGAAATTTCTCAGCAGCAT	-	c.576_577ins, GAATGCTGCTGAGAAATTTCTTGCCAAATTTAGTAATATTTCTCCAGAGAACCACATTTCT	p.T193Efs*4	0.0005	0.0004	.	.	D	—	1	0
FRG1	4q35.2	A	G	c.G322A	p.A108T	0	0	D	P	D	—	13	0
FRG1	4q35.2	-	AAG	c.631_633del	p.K212del	0	0	.	.	D	—	6	1
SDHA	5p15.33	C	T	c.T1193C	p.V446A	0	0	D	D	D	—	11	2
SDHA	5p15.33	T	C	c.C1202T	p.A449V	0	0	D	D	D	—	11	3
NUP153	6p22.3	A	T	c.A938T	p.Q313L	0.0014	0.0021	D	D	D	—	1	0
HLA-DRB	6p21.32	A	T	c.A242T	p.E81V	0	0	D	D	D	—	1	1
BCLAF1	6q23.3	A	C	c.G807T	p.R271S	0	0	D	D	D	—	1	0
BCLAF1	6q23.3	T	G	c.C487A	p.Q165K	0.0001	0.0002	D	P	D	—	1	0
PABPC1	8q22.3	G	A	c.T1685C	p.L562S	0.0007	0.0036	D	D	D	—	3	0
CTBP2	10q26.13	T	C	c.G871A	p.V223M	0	0	D	D	D	—	14	0
CTBP2	10q26.13	A	G	c.C749T	p.T185M	0	0.00005459	D	D	D	—	1	0
CTBP2	10q26.13	T	C	c.G553A	p.G117S	0	0	D	D	D	—	8	0
CTBP2	10q26.13	A	T	c.A22T	p.K8X	0	0	.	.	A	—	17	0
ARHGAP	14q12	C	T	c.T1421C	p.V474A	0.0006	0	D	P	D	—	2	0
CDC27	17q21.32	T	C	c.G1486A	p.A496T	0	0	D	D	D	—	17	0
CNN2	19p13.3	A	G	c.G670A	p.G263S	0	0	D	D	D	—	14	14
CNN2	19p13.3	A	G	c.G680A	p.R266Q	0	0.0001	D	P	D	—	14	14

NA change[a], nuclear acid change; AA change[b], amino acid change; EAS[c], East Asian; SIFT (D: damaging); Polyphen-2 (D: damaging, P: pathogenic); Mutation Taster (D: disease causing, A:disease causing); dbSNP[d], Single Nucleotide Polymorphism database

Table S7

Prediction of free energy change (ΔΔG), conservation and non-covalent force of mutation sites

Gene	NA Change[a]	AA Change[b]	Predicted ΔΔG(kcal/mol)	conservation	H Bond change[c]	Polar groups
SDHA	c.T1337C	p.V446A	0.25(Increase Stability)	high	—	non-polar→non-polar
SDHA	c.C1346T	p.A449V	0.98(Increase Stability)	high	—	non-polar→non-polar
BCLAF1	c.G813T	p.R271S	-1.88(Decrease Stability)	high	—	positively charged→polar noncharged
BCLAF1	c.C493A	p.Q165K	-0.36(Decrease Stability)	high	—	polar noncharged→positively charged
PABPC1	c.T1685C	p.L562S	-1.51(Decrease Stability)	high	1	non-polar→polar noncharged
CTBP2	c.G667A	p.V223M	-1.39(Decrease Stability)	high	—	non-polar→non-polar
CTBP2	c.C545T	p.T182M	-0.24(Decrease Stability)	high	—	polar noncharged→non-polar
CTBP2	c.G349A	p.G117S	-2.11(Decrease Stability)	high	4	polar noncharged→polar noncharged
CNN2	c.G787A	p.G263S	-0.99(Decrease Stability)	high	—	polar noncharged→polar noncharged
CNN2	c.G797A	p.R266Q	-0.42(Decrease Stability)	high	—	positively charged→polarnoncharged

NA change[a], nuclear acid change; AA change[b], amino acid change; H Bond change[c], hydrogen bonding change
